# Estimation of Fast and Slow Adaptions in the Tactile Sensation of Mechanoreceptors Mimicked by Hybrid Fluid (HF) Rubber with Equivalent Electric Circuits and Properties

**DOI:** 10.3390/s23031327

**Published:** 2023-01-24

**Authors:** Kunio Shimada

**Affiliations:** Faculty of Symbiotic Systems Sciences, Fukushima University, 1 Kanayagawa, Fukushima 960-1296, Japan; shimadakun@sss.fukushima-u.ac.jp; Tel.: +81-24-548-5214

**Keywords:** mechanoreceptor, tactile sensation, firing rate, equivalent electric circuit, electric property, mimesis, slow adaption (SA), fast adaption (FA), rubber, electrolytic polymerization, hybrid fluid (HF)

## Abstract

In order to advance engineering applications of robotics such as wearable health-monitoring devices, humanoid robots, etc., it is essential to investigate the tactile sensations of artificial haptic sensors mimicking bioinspired human cutaneous mechanoreceptors such as free nerve endings, Merkel’s cells, Krause end bulbs, Meissner corpuscles, Ruffini endings, and Pacinian corpuscles. The generated receptor’s potential response to extraneous stimuli, categorized as slow adaption (SA) or fast adaption (FA), is particularly significant as a typical property. The present study addressed the estimation of SA and FA by utilizing morphologically fabricated mechanoreceptors made of our proposed magnetically responsive intelligent fluid, hybrid fluid (HF), and by applying our proposed electrolytic polymerization. Electric circuit models of the mechanoreceptors were generated using experimental data on capacitance and inductance on the basis of the electric characteristics of impedance. The present results regarding equivalent firing rates based on FA and SA are consistent with the FA and SA findings of vital mechanoreceptors by biomedical analysis. The present investigative process is useful to clarify the time of response to a force on the fabricated artificial mechanoreceptor.

## 1. Introduction

Robotic techniques that have the potential to enrich our lives have become essential and pervade many aspects of industry, space exploration, rehabilitative therapeutics, etc. [1]. There has been particular focus on the medical robotics used in prosthetic rehabilitation devices that have human–machine interfaces for their challenged users, on autonomous driving robots, and on substitutions for human organs or tissues for physically impaired patients [2]. The achievement of these targets requires certain prerequisite conditions of conformability; for example, flexibility and elasticity are essential in artificial human organs and tissues in order for them to meet the requirements of both safety and comfort. In the case of artifacts that are required to carry out functions comfortably, such as providing a secure and hospitable environment, it is often best to adapt the morphological paradigm to mimic the human body, for example, cutaneous mechanoreceptors in the human skin are responsible for tactile sensation [3]. The other senses are experienced through other receptors: olfactory and gustatory sensations through chemoreceptors, vision through photoreceptors, thermal sensation through thermoreceptors. Bioinspired artificial organs and tissues can be advanced by mimicking mechanoreceptors to integrate electronic systems such as piezo-resistivity, piezo-electricity, piezo-capacity, and/or tribo-electricity [2]. The electric signal from the mimicked artificial sensor provides the sensory cognition that interprets the haptic bio-impulses. The artificial bioinspired mechanoreceptors must be made of a soft material, such as rubber or sponge, or synthetic materials such as elastomers or polymers, in order to allow large elastic deformation and strain [4]. This view is supported by the typical findings of research on electronic skin (e-skin) [5,6], etc. Additionally, it is important to simulate the characteristics of tactile mechanoreceptors such as free nerve endings, Merkel’s cells, Krause end bulbs, Meissner corpuscles, Ruffini endings and Pacinian corpuscles [7,8]. The generated receptor’s potential response to extraneous stimuli, categorized as slow adaption (SA) or fast adaption (FA), is also a typical property [9,10]. SA and FA are based on ion channel systems with electric circuits that mimic the human skin’s sensory abilities [11]. Furthermore, the ion channel systems provide the bioinspired electronics that respond to mechanical stimuli such as normal force, shear motion, and vibration.

We previously proposed flexible and elastic mechanoreceptors made of rubber, which can accomplish large elongation and compression as well as bending, in contrast to other artificial mechanoreceptors made of solid membranes and substrates such as e-skin [12]. For tactile sensation in particular, self-powered mechanoreceptors using a hybrid fluid (HF) as a magnetically responsive intelligent fluid and a novel rubber solidification technique using electrolytic polymerization have been investigated for their response to a variety of external stimuli such as pressure, thermal condition, etc. [13]. However, the instantaneous responsive FA and SA functions of our proposed mechanoreceptors have not yet been elucidated. Their clarification could advance the development of many applications of robotics such as wearable health-monitoring devices, humanoid robots, etc. Therefore, the purpose of the present study was to calculate the mechanical responses based on FA and SA with equivalent electric circuits electrically stimulating mechanoreceptors which are induced from their electric properties.

## 2. Materials

**Figure A1:**
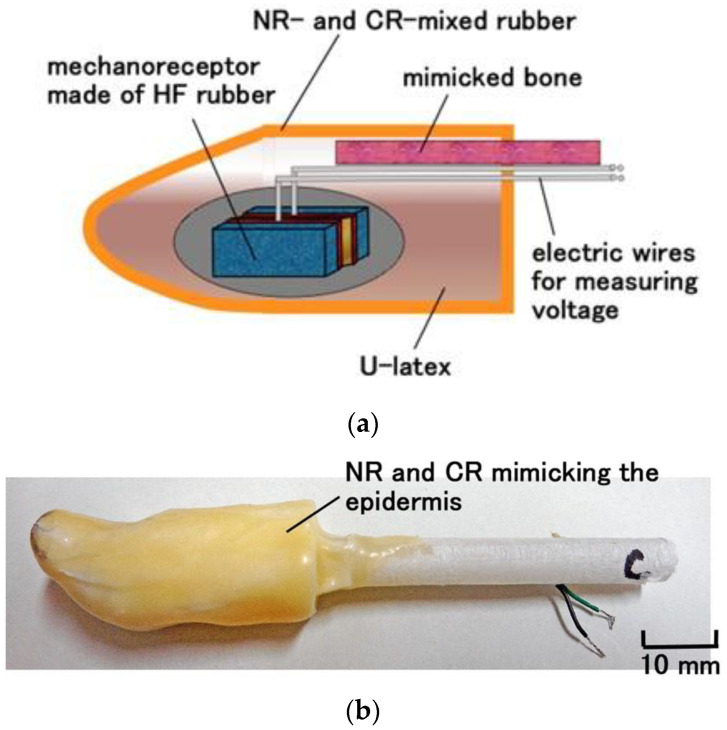
Fabricated finger embedded with a mechanoreceptor: (**a**) schematic cross-section; (**b**) image of a fabricated finger [13].

The mechanoreceptors dealt with in the previous and present studies [12,13] are free nerve endings (Type A), Krause end bulbs (Type B), Meissner corpuscles (Type C), Pacinian corpuscles (Type D) and Ruffini endings (Type E), which were embedded in a thumb-shaped molded finger made of urethane rubber (U; Human skin gel, 0-solidity; Exseal Co., Ltd., Gihu, Japan) with 1.875 × 10^−6^ -Pa^−1^ compressibility. The fabricated finger was coated with a combination of natural rubber (NR; Ulacol; Rejitex Co., Ltd., Atsugi, Japan) and chloroprene rubber (CR; 671A; Showa Denko Co., Ltd., Tokyo, Japan), a mixed rubber that mimics the human epidermis, as shown in Figure A1 in Appendix A. The mechanoreceptors were fabricated by our proposed state-of-the-art rubber-solidification technique, which provides the following novel developments in rubber engineering [12]. These developments are achieved through electrolytic polymerization.

a. Novel solidification of a rubber: rubber including water can be vulcanized by the application of an electric field so that the molecules of the rubber can be crosslinked, which is different from ordinary vulcanization with sulfur; the involvement of water makes solidification possible by mixing NR or CR rubber.
b. Production of pores and infiltration of a liquid into the rubber: a rubber can have many pores due to electrolytic polymerization with mixing in a metallic hydrate such as Na_2_WO_4_ 2H_2_O.
c. Adhesion of the rubber to a metal: through electrolytic polymerization and the addition of water and a metallic hydrate such as Na_2_WO_4_ 2H_2_O, we can adhere the rubber to a metal; the electric wires adhered to the sensor as electrodes to measure voltage need to adhere to the rubber to prevent them from detaching.

We create the fabricated mechanoreceptors by mixing HF in rubber and utilizing the electrolytic polymerization technique, which is discussed in greater detail in our previous study [13]. HF is an optimal fluid for electrolytic polymerization of rubber, and contains water, kerosene, silicon oil, polyvinyl alcohol (PVA), Fe_3_O_4_ particles, Fe particles approximately 50-μm in size, and sodium hexadecyl sulfate aqueous solution for surfactant [13]. HF rubber consists of HF, NR and CR rubbers and μm-order Ni particles. The electrolytic polymerization is conducted under the application of a magnetic field such that magnetic clusters structured by the metal particles are created to be aligned along the direction of the applied magnetic field. The alignment of the magnetic clusters induces the enhancement of magnetic and mechanical strengths, and of electric and thermal conductivities.

## 3. Methods

In order to estimate the response to mechanical stimuli of these mechanoreceptors, we used the same mechanical apparatus to investigate the application of a normal force (normal force experiment, NFE) [13]. The finger was moved in hot or cold water, continuously touching the bottom of the vessel. The up and down motion in the water was repeated a few times by a compression testing machine (SL-6002; IMADA-SS Co., Ltd., Toyohasi, Japan) at a velocity of 300 mm/min. The voltage from the receptor in the thumb was measured using a voltage meter (PC710; Sanwa Electric Instrument Co., Ltd., Tokyo, Japan).

The electric properties of the mechanoreceptors were measured using an LCR meter (IM3536; Hioki Co., Ltd., Ueda, Japan). These electric properties can be evaluated by the behavior of the resistance in the alternating current (AC) circuits, in which the time-varying current is generated. Not only the electric structure of the material but also the time-changing tendency of the potential can be estimated from the resistance, determined as the opposition to the time-varying current.

## 4. Results and Discussion

### 4.1. Inner Electrical Phenomena

**Figure 1:**
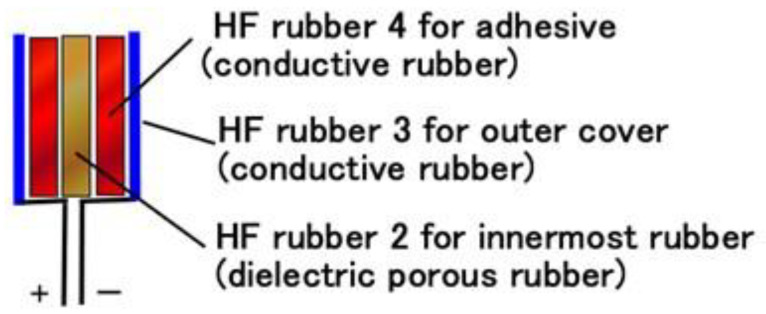
The structure of the primary component unit of HF-rubber mechanoreceptors.

**Figure A2:**
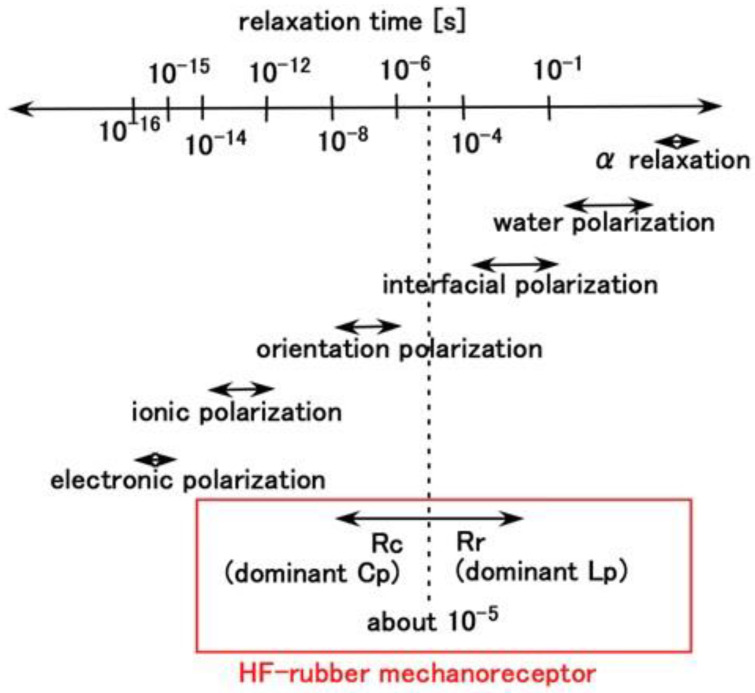
Schematic of the categories of polarization relaxation time of a dielectric material.

The primary component units of the fabricated mechanoreceptors were created with HF rubbers 2–4, which have different constitutions, as discussed in our previous studies [12,13] and as shown in Figure 1. HF rubber 2 is a permeable rubber made using Na_2_WO_4_ H_2_O. As it is porous, it is permeable by any liquid. In the present study, glycerin was added to the rubber in order to make the rubber a capacitor. HF rubber 3 was used for the outer cover of the sensor. HF rubber 4, which is made with both water and Na_2_WO_4_ 2H_2_O, serves as an adhesive between HF rubbers 2 and 3, and between the HF rubber 4 and the electric wire. Because HF rubber is piezoresistive so that it can be conductive, as shown in our previous study [12], HF rubbers 3 and 4 are conductive. Thus, the mechanoreceptors are similar to an electrolytic capacitor and there is polarization. This can also be seen from the viewpoint of the involvement of NR. Because the composite material consisting of the NR matrix has polarization, three polarization relaxation processes take place—α dipolar relaxation, water polarization relaxation, and interfacial polarization [14,15]—as does a conduction phenomenon, as shown in Figure A2 in Appendix B.

**Figure 2:**
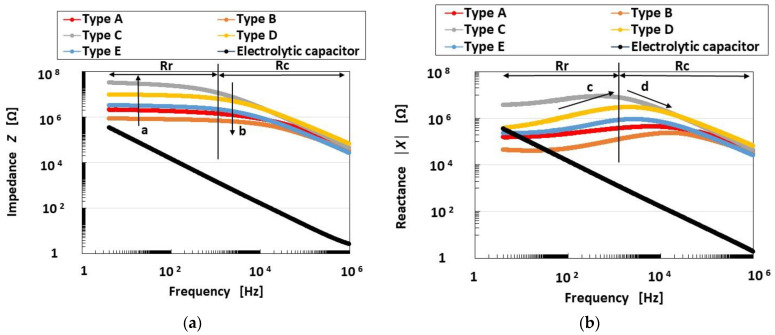
Impedance and reactance of the mechanoreceptors: (**a**) impedance; (**b**) reactance.

**Figure 3:**
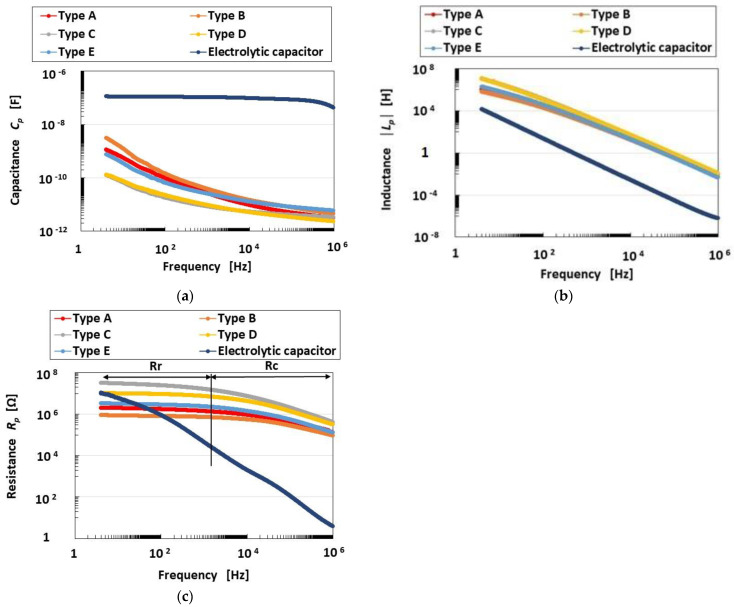
Capacitance, inductance and resistance of the mechanoreceptors: (**a**) capacitance; (**b**) inductance; (**c**) resistance.

**Figure 4:**
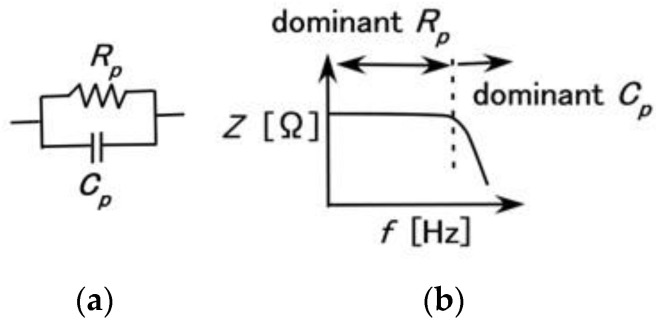
The tendency of impedance based on a parallel electric circuit: (**a**) electric circuit; (**b**) change of impedance based on the parallel electric circuit.

Figure 2 shows the measurement results of the impedance *Z* and reactance *X* of the mechanoreceptors, and Figure 3 shows those of capacitance *C_p_*, inductance *L_p_* and resistance *R_p_*. These results are compared with those of an ordinary electrolytic capacitor (A0830, 0.1 μF, 100 V). As the impedance is in the range of 10^4^ to10^8^, conductance *G_p_* and susceptance *B_p_* are able to form a parallel circuit. Therefore, the mechanoreceptor can be assumed to have predominantly a primary unit consisting of a parallel-type electric circuit structured by resistance and capacitance, as shown in Figure 4a. On the other hand, from the result that *Z* holds almost constant in range Rr, but decreases with increasing frequency in range Rc (Figure 2a), *Z* presents the qualitative tendency demonstrated by *R_p_* and *C_p_*, as shown in Figure 4b. Here, as with other tendencies, we must be careful to ensure that *R_p_* remains comparatively large over the frequency range. Based on the results of *Z* and *X*, *R_p_* and *C_p_* in the mechanoreceptor can be seen as follows.

In range Rr, *R_p_*, which holds almost constant, dominates in the mechanoreceptor, rather than *C_p_*. On the other hand, in range Rc, *C_p_* dominates, rather than *R_p_*. Therefore, *R_p_* is large in range Rr and decreases in range Rc. The tendency of *R_p_* in range Rr and its decrease with increasing frequency in range Rc coincides with that shown in Figure 3c. As for *C_p_*, decreasing *Z* means decreasing *C_p_* with the result that *C_p_* decreases with increasing frequency. The tendency of *C_p_* to decrease with increasing frequency coincides with that shown in Figure 3a.

Increasing *Z* means increasing *R_p_*, as shown by the up-arrow a in range Rr in Figure 2a. Therefore, *R_p_* decreases in Types C, D, E, A, and B, in that order. As for *C_p_*, decreasing *Z* means increasing *C_p_*, as shown by the down-arrow b in range Rc in Figure 2a. Therefore, *C_p_* decreases in Types C, D, E, A, and B, in that order. Incidentally, as a result, the qualitatively changing tendency of *R_p_* is converse to that of *C_p_*.

In the case of *X*, there are also two ranges, Rr and Rc, as shown in Figure 2b. The tendency of *X* involves the effect of *L_p_* in contrast to that of *Z*: *L_p_* dominantly affects Z in range Rr so that *L_p_* increases with increasing *X*; *C_p_* dominantly affects *Z* in range Rc so that *C_p_* increases with decreasing *X*. *X* increases in range Rr and decreases in range Rc with increasing frequency, as shown by the up-arrow c and down-arrow d, respectively, in Figure 2b. By transmitting from range Rr to range Rc, the effect of *L_p_* decreases. This tendency coincides with that shown in Figure 3b. Increasing *X* means increasing *L_p_*, as shown by the up-arrow c in range Rr in Figure 2b. Therefore, *L_p_* decreases in Types C, D, E, A, and B, in that order. In contrast, decreasing *X* means increasing *C_p_*, as shown by the down-arrow d in range Rc in Figure 2b. Therefore, *C_p_* increases in Types C, D, E, A, and B, in that order. Incidentally, as a result, the qualitatively changing tendency of *L_p_* is the same as that of *R_p_*.

In conclusion, based on the above results (Figure 2 and Figure 3), the electrical phenomena in the present HF-rubber mechanoreceptors can be estimated as follows, by supplementing Figure A2 in Appendix B.

The frequency at the boundary between Rr and Rc is approximately 5 kHz (Figure 2 and Figure 3c). In range Rr, not only the ions of the water involved in the HF rubber but also the negative and positive charges on the boundary between the differential kinds of particles and molecules of the HF rubber based on an electric double layer, are polarized, with the result that *L_p_* becomes dominant. On the other hand, in range Rc, the ions and the permanent dipoles of the particles and molecules of the HF rubber are polarized such that *C_p_* becomes dominant. Over the ranges of Rr and Rc, resistance *R_p_* is large.

In addition, in comparison with that of the ordinary electrolytic capacitor, the *C_p_* of the mechanoreceptor is smaller, but its *L_p_* and *R_p_* are larger than those of the ordinary electrolytic capacitor.

### 4.2. Equivalent Electric Circuit

**Figure 5:**
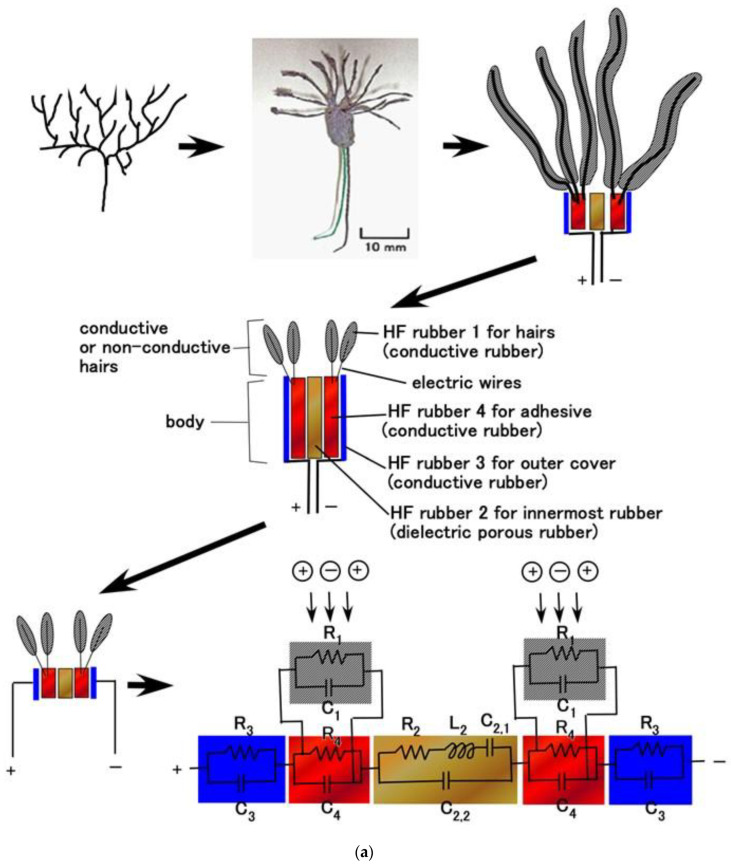

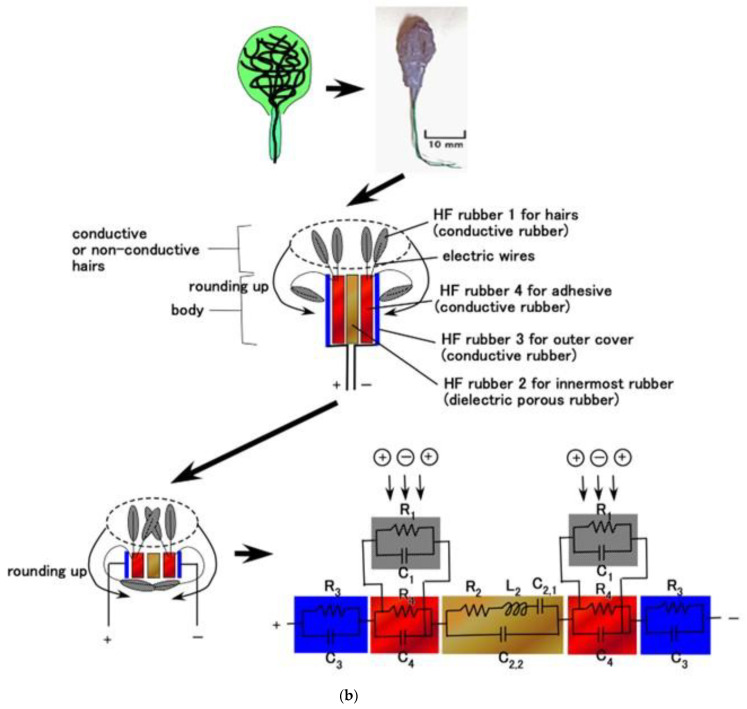

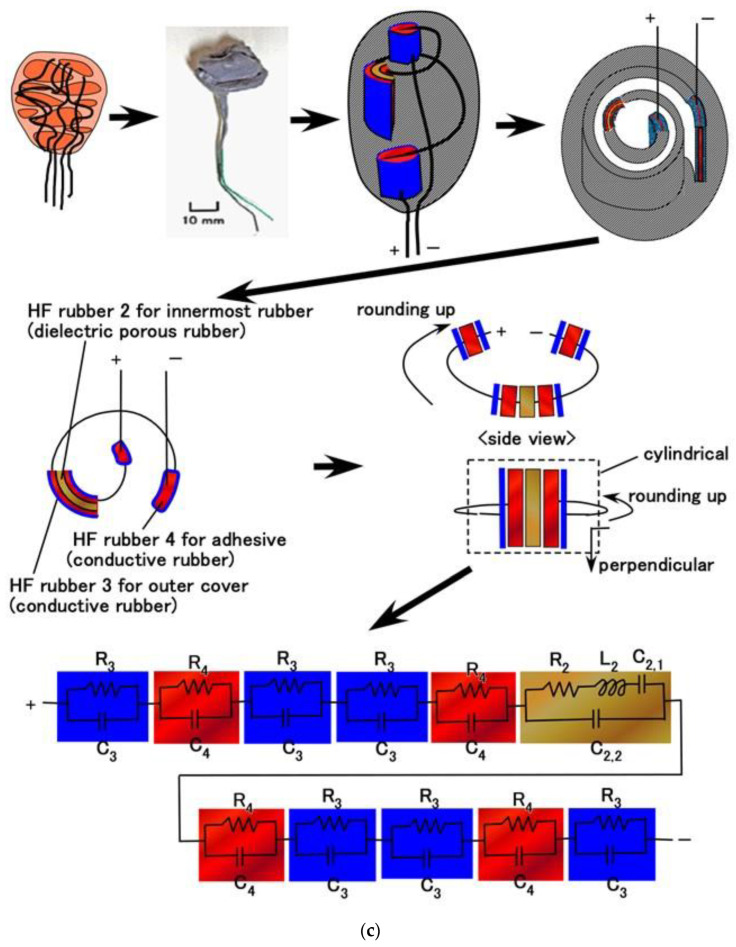

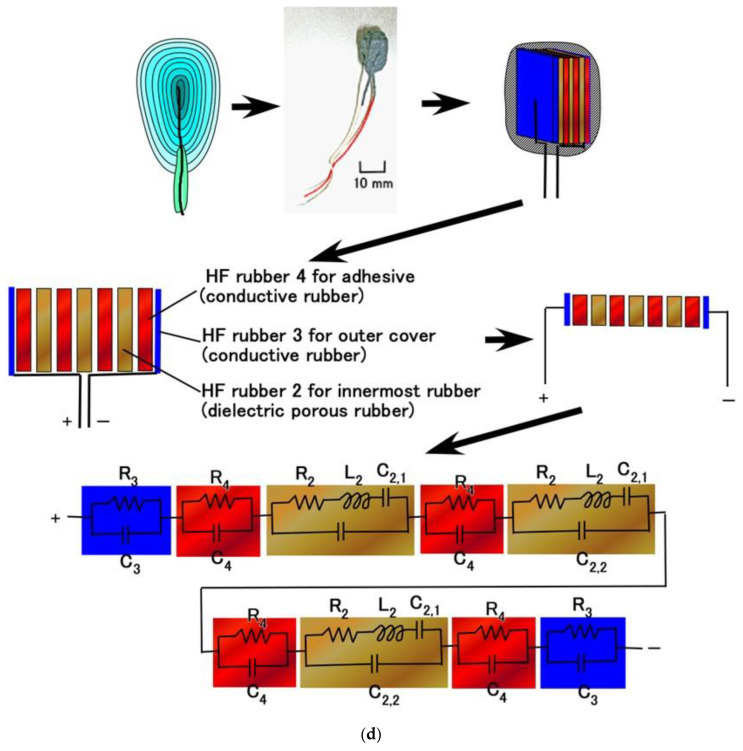

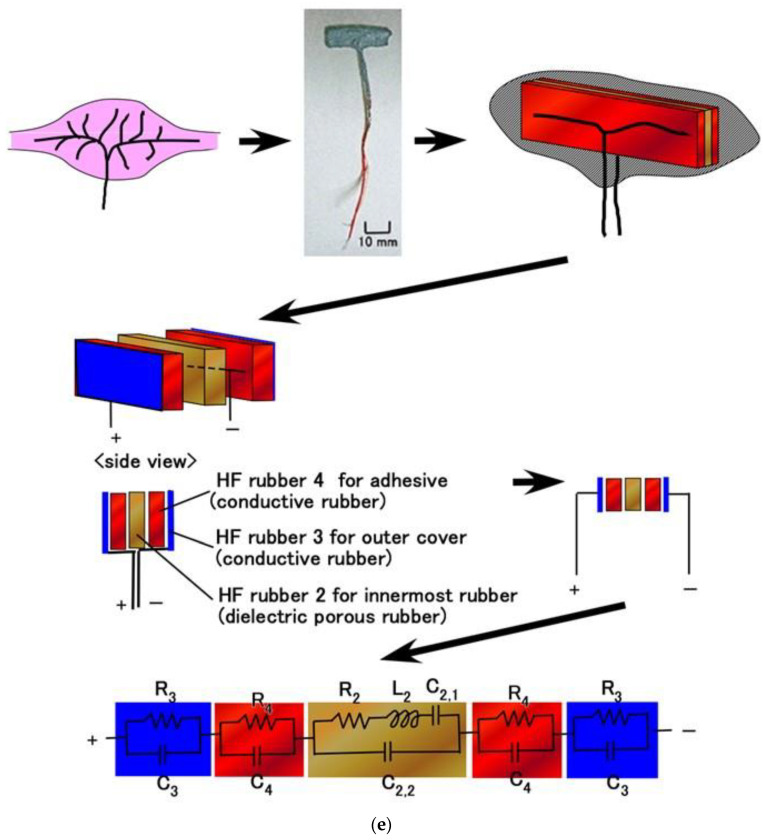
Schematic of our cutaneous mechanoreceptor, images of the fabricated mechanoreceptor, schematic of the fabricated mechanoreceptor, structure of the fabricated mechanoreceptor, equivalent electric circuit: (**a**) free nerve endings; (**b**) Krause end bulbs; (**c**) Meissner corpuscles; (**d**) Pacinian corpuscles; (**e**) Ruffini endings.

Based on the primary unit of the electric circuit structured as a parallel circuit by resistance and capacitance (Figure 4a), we demonstrate the equivalent electric circuit of the HF-rubber mechanoreceptors (Figure 5). HF rubber 2 becomes a condenser as dielectric material including *R_p_*, *C_p_*, and *L_p_*, and HF rubbers 1, 3, and 4 conductive materials including *R_p_* and *C_p_*, whose electrical model was enunciated by a self-powered sensor mimicking cutaneous mechanoreceptors in another study on e-skin [12]. Each panel of Figure 5 presents a schematic of the virtual mechanoreceptor, our fabricated HF-rubber mechanoreceptor, a schematic of the inner structure of the fabricated mechanoreceptor, and the equivalent electric circuit based on the schematic of the inner structure.

### 4.3. Firing Rate

**Figure A3:**
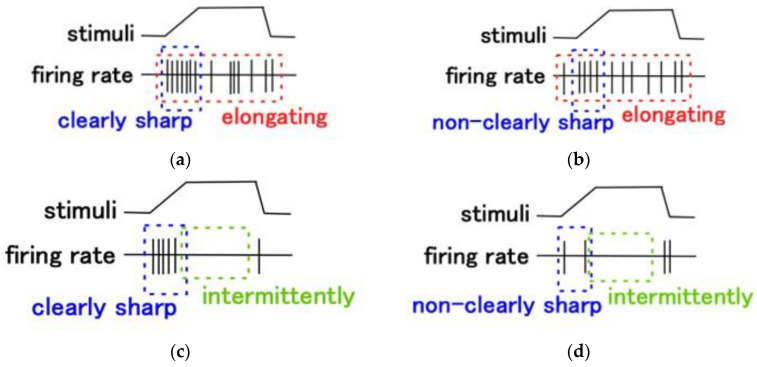
FA and SA of the vital mechanoreceptors by biomedical analysis: (**a**) SA I; (**b**) SA II (**c**) FA I; (**d**) FA II.

When the skin responds to extraneous stimuli, mechanoreceptors in the skin generate changes in the potential as electric impulses. By means of the active potential, SA and FA are inspired as abrupt stimuli. They are generally categorized as SA I, SA II, FA I, and FA II, as shown in Figure A3 in Appendix C. For example, based on the classification of mechanoreceptors, Merkel disks correspond to SAnI, Ruffini endings to SA II, Meissner corpuscles to FA I, and Pacinian corpuscles to FA II [16,17]. The stimuli at the neuron in the human body is presented as the action potential created by the instantaneous influx of sodium ions (Na^+^), and manifests as the neuron firing. The frequency of firing is defined as the firing rate [18]. The timing of the firing, evidenced by spikes in the potential, is significant but independent of the magnitude or shape of the peak. Therefore, the firing rate λ(*t*) is the differentiation of the mean spike count *N* (*t*), as shown in Equation (1), where *t* is the time and ε is the diminutive increment of *t*. Thus, the firing rate can be estimated as the gradient of the changing potential, which has been shown by research demonstrating that the firing rate can be calculated from mathematically estimated equations using firing rate models for FA and SA [19].

(1) $$\lambda \left(t\right)=\underset{\varepsilon \to 0}{\lim}\frac{N\left(t,t+\varepsilon \right)}{\varepsilon }$$

**Figure 6:**
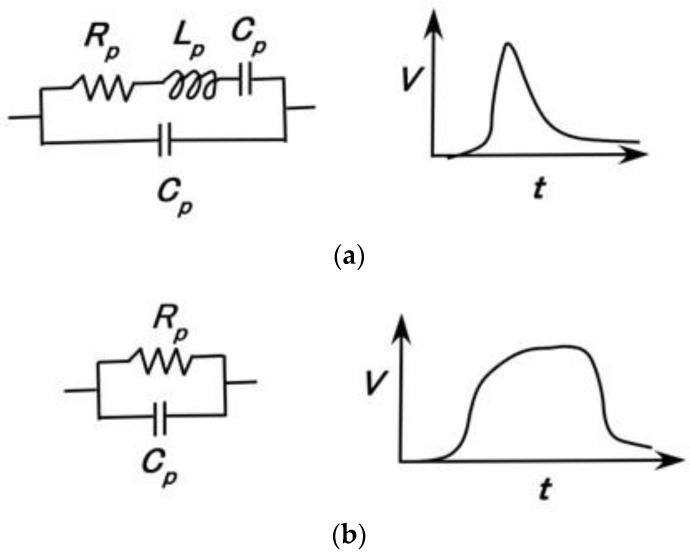
Changes in potential based on FA (**a**) and SA (**b**).

The firing rate is strongly related to *C_p_* and *L_p_* under the assumption of the equivalent electric circuit. FA depends on *L_p_*, yielding a potential that changes rapidly and has a peak, as shown in Figure 6a, while SA depends on *C_p_*, yielding a potential that changes smoothly or gradually, as shown in Figure 6b. Specifically, the electric circuit as delineated in the left figure characterizes the change of the potential as shown in the right figure, respectively, in Figure 6. The data mathematically calculated from the potential changes corresponds to the firing rate. Although it does not demonstrate the absolute value of the firing rate, it is directly proportional to the firing rate so that it is defined by the equivalent firing rate.

**Figure 7:**
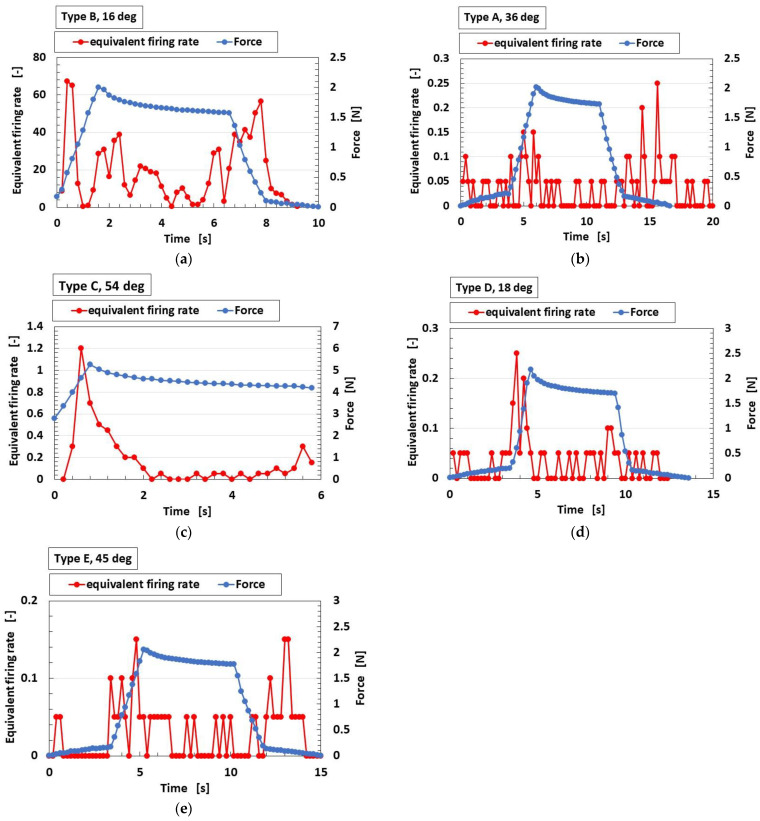
Equivalent firing rate to applied normal force: (**a**) free nerve endings; (**b**) Krause end bulbs; (**c**) Meissner corpuscles; (**d**) Pacinian corpuscles; (**e**) Ruffini endings.

The equivalent firing rate, determined from the measured data of the potential of the fabricated finger with the mechanoreceptor upon the application of normal force, is shown in Figure 7. The temperature of the water in the vessel is also shown in the figure. The potential of the mechanoreceptor is the built-in voltage generated from the self-powered HF rubber, as demonstrated in our previous studies [12,13]. The equivalent firing rate is obtained by the differential of the built-in voltage, such that it is estimated as the proportionality of the firing rate, as mentioned above.

Types C and D show a typical peak whose equivalent firing rate changes abruptly and clearly. However, Types A, B and E show multiple peaks with the same quantitative magnitude, whose equivalent firing rate cannot be seen clearly. Therefore, Types C and D correspond to FA, and Types A, B and E to SA.

### 4.4. FA and SA

**Figure 8:**
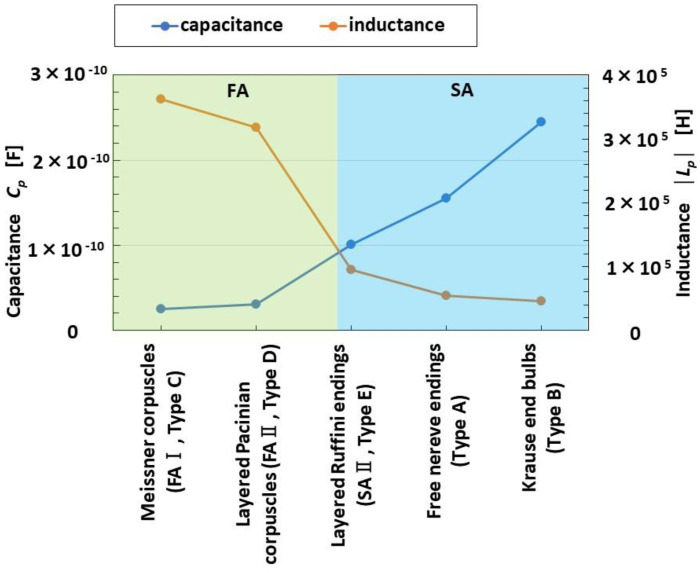
Relations among fabricated mechanoreceptors, capacitance and inductance.

The *C_p_* and *L_p_* data shown in Figure 3 allow Types A-E to be arranged in a line. Taking this together with the fact that the effect of *L_p_* brings about FA and the effect of *C_p_* brings about SA as shown in Figure 6, we can organize our results as shown in Figure 8. Furthermore, we can also classify Types A-E as corresponding to either FA or SA, as shown in Figure 7.

In the fabrication of Type A, the tip of HF rubber 1 has agglomeration for hair, as shown in our previous study [12]. Therefore, the electric circuit of Type A makes it equivalent to Merkel disks, and Type A are thought to correspond to SA, which is in order next to SA II. As a result, Types A and B are drawn up in SA I. These results are consistent with the findings of the order of FA and SA in vital mechanoreceptors by biomedical analysis.

From the result that increasing *X* means increasing *L_p_*, as shown by the up-arrow in range Rr in Figure 2b, in Types C, D, E, A, and B, in that order, the response occurs slowly, so that these types lined up horizontally at the abscissa in Figure 8. In contrast, from the result that decreasing *X* means increasing *C_p_*, as shown by the down-arrow in range Rc in Figure 2b, in Types C, D, E, A, and B, in that order, again, the response occurs slowly.

## 5. Conclusions

Our previous studies dealt with the time-varying response to an applied force from the morphological perspective of fabricated HF-rubber mechanoreceptors. In contrast, the present study addresses the same issue, but from an electrical perspective using electric properties. The electric circuit and the firing rate of the mechanoreceptor can be estimated from the data of impedance, reactance, etc., to determine FA and SA as responsive stimuli. The identified FA and SA results are consistent with the FA and SA findings on vital mechanoreceptors using biomedical analysis. The present process is a novel method to identify the electrical model of a mechanoreceptor.

The present investigation of the instantaneous response to a force could advance the development of many applications of robotics such as wearable health-monitoring devices, humanoid robots, etc. It is therefore highly useful to investigate the time response to a force on a fabricated artificial mechanoreceptor.

## Data Availability

Not applicable.

## Appendix A

Figure A1 shows the mimicked finger embedded with a mechanoreceptor and shaped like a thumb. This finger was used in the experiment to test the response to a normal force.

## Appendix B

In general, a dielectric material is polarized by the application of an extraneous electric field. The polarization relaxation time is categorized according to several phenomena of dielectric polarization, as shown in Figure A2 [14,15], which includes a comparison with the present HF-rubber mechanoreceptors.

## Appendix C

The ordinary findings of the FA and SA of the vital mechanoreceptors using biomedical analysis are shown in Figure A3.

## References

[1] Yang G.Z., Bellingham J., Dupont P.E., Fischer P., Floridi L., Full R., Jacobstein N., Kumar V., McNutt M., Merrifield R. (2018). The grand challenges of science robotics. Sci. Robot..

[2] Heng W., Solomon S., Gao W. (2022). Flexible electronics and devices as human-machine interfaces for medical robotics. Adv. Mater..

[3] Jung Y.H., Park B., Kim J.U., Kim T. (2019). Bioinspired electronics for artificial sensory systems. Adv. Mater..

[4] Li S., Bai H., Shepherd R.F., Zhao H. (2019). Bio-inspired design and additive manufacturing of soft materials, machines, robots, and haptic interfaces. Angew. Chem. Int. Ed..

[5] Yang J.C., Mun J., Kwon S.Y., Park S., Bao Z., Park S. (2019). Electronic skin: Recent progress and future prospects for skin-attachable devices for health monitoring, robotics, and prosthetics. Adv. Mater..

[6] Chortos A., Liu J., Bao Z. (2016). Pursuing prosthetic electronic skin. Nat. Mater..

[7] Munger B.L., Ide C. (1988). The structure and function of cutaneous sensory receptors. Arch. Histol. Cytol..

[8] French A.S., Torkkeli P.H., Squire L.R. (2009). Mechanoreceptors. Encyclopedia of Neuroscience.

[9] Hamann W. (1995). Mammalian cutaneous mechanoreceptors. Prog. Biophys. Mol. Biol..

[10] Bolanowski S.J., Gescheider G.A., Verrillo R.T., Checkosky C.M. (1988). Four channels mediate the mechanical aspects of touch. J. Acoust. Soc. Am..

[11] Chun K.Y., Son Y.J., Jeon E.S., Lee S., Han C.S. (2018). A self-powered sensor mimicking slow- and fast-adapting cutaneous mechanoreceptors. Adv. Mater..

[12] Shimada K. (2022). Morphological conFigureuration of sensory biomedical receptors based on structures integrated by electric circuits and utilizing magnetic-responsive hybrid fluid (HF). Sensors.

[13] Shimada K., Ikeda R., Kikura H., Takahashi H. (2021). Morphological fabrication of rubber cutaneous receptors embedded in a stretchable skin-mimicking human tissue by the utilization of hybrid fluid. Sensors.

[14] Ladhar A., Arous M., Kaddami H., Raihane M., Kallel A., Graca M.P.F., Costa L.C. (2015). AC and DC electrical conductivity in natural rubber/nanofibrillated cellulose nanocomposites. J. Mol. Liq..

[15] Ladhar A., Arous M., Kaddami H., Raihane M., Lahcini M., Kallel A., Graca M.P.F., Costa L.C. (2013). Dielectric relaxation studies on nanocomposites of rubber with nanofibrillated cellulose. J. Non-Crys. Solids.

[16] Wang Q., Fan C., Gui Y., Zhang L., Zhang J., Sun L., Wang K., Han Z. (2021). Engineered mechanosensors inspired by biological mechanosensilla. Adv. Mater..

[17] Tiwana M.I., Redmond S.J., Lovell N.H. (2012). A review of tactile sensing technologies with applications in biomedical engineering. Sens. Actuators A Phys..

[18] Tomar R. (2019). Review: Methods of firing rate estimation. BioSystems.

[19] Nezhad N., Amiri M., Falotico E., Laschi C. (2018). A digital hardware realization for spiking model of cutaneous mechanoreceptor. Fron. Neuro..

